# Actions Targeting the Double Burden of Malnutrition: A Scoping Review

**DOI:** 10.3390/nu12010081

**Published:** 2019-12-27

**Authors:** Sonia Menon, José L. Peñalvo

**Affiliations:** 1 Unit of Noncommunicable Diseases, Department of Public Health, Institute of Tropical Medicine, Nationalestraat, 155 2000 Antwerp, Belgium; smenon@itg.be; 2 Friedman School of Nutrition Science and Policy, Tufts University, Boston, MA 02111, USA

**Keywords:** double burden of malnutrition, non communicable diseases, double duty actions

## Abstract

Background: In many developing countries, nutritional and epidemiological transitions are contributing to continuous undernutrition and escalating overnutrition, resulting in coexisting forms of malnutrition often referred as the “double burden of malnutrition” (DBM). This complex phenomenon constitutes an unprecedented challenge to global public health and has been prioritized by international health organizations, prompting governments to swift action. Specifically, five years ago the World Health Organization (WHO) proposed a roadmap to tackle the DBM though so-called “double-duty actions”. The objective of this review was to synthesize the literature on interventions which address the DBM. Methods: We developed a scoping review to identify interventions addressing the DBM. We searched PUBMED for papers reporting interventions until December 2019. Articles examining interventions, government policies, or tools at the individual, household, or community level to address the DBM were included. Results: Seven articles met the inclusion criteria. Three were from sub-Saharan Africa, one was from Southeast Asia, and one was from Central America. Two were modelling studies, with one covering 24 low-income countries and the other focusing on Ghana. Conclusion: Notwithstanding the pressing issue of the DBM, there is a paucity of studies examining double-duty actions despite the attention that it has garnered within the global nutrition community. Whilst nutrient deficiencies may be curbed by poverty reduction measures, for obesity prevention nutrition, education and promotion of physical activity, along with the encouragement of local food production, may be instrumental.

## 1. Introduction

In 2016, approximately 1.9 billion adults were estimated to be overweight or obese, while 462 million were underweight [1]. In 2016, WHO estimated the global number of overweight children aged under five to be over 41 million, of which almost half lived in Asia and one quarter in Africa [2]. Wasting continues to threaten the lives of an estimated 7.7% or nearly 52 million children under 5 years globally. Stunting affected an estimated 22.9% or 154.8 million children under 5 years of age globally in 2016, coexisting with an unprecedented increase in overweight and obesity in the past few years in many of these populations, giving rise to the term “double burden of malnutrition” (DBM) [3]. This dual burden can manifest at the individual, household, or population levels. At the individual level it occurs with the emergence of two or more forms of malnutrition observed simultaneously—undernutrition represented by wasting, stunting, or micronutrient deficiencies co-occurring with overnutrition overweight/obesity or subsequent overweight in an undernourished child. At the household level, the DBM may arise from contrasting forms of malnutrition among different family members. Socio-economic status heavily influences the extent of the dual burden, with obesity increasingly affecting the already undernourished poor. Finally, as a reflection of the accelerated nutritional, demographic, and epidemiological transitions in developing countries at the population level, both undernutrition and overweight are prevalent in the same communities, regions, or nations [3].

Whilst the worldwide rollout of conditional cash transfer programs to households on the condition that they comply with certain behavioural conditions as strategies to reduce poverty have been yielding a positive impact on child undernutrition [4], in 2016, the United Nations Decade of Action on Nutrition for the period 2016–2025 came to life, calling for specific coordinated actions through cross-cutting and coherent policies, programs, and initiatives including social protection to comprehensively simultaneously address increasing DBM. As the global community transitions from a predominant focus on the eradication of severe and acute undernutrition within the framework of the Millennium Development Goals to the broader nutrition focus of the Sustainable Development Goals (SDG)—including malnutrition in all its forms and noncommunicable diseases—the emergence of DBM offers an untapped window of opportunity for integrated actions, generally termed “double-duty actions” (DDAs). In 2017, WHO published a first set of five potential DDAs (Table 1) including interventions on the promotion of exclusive breastfeeding and actions to optimize early nutrition, maternal nutrition, antenatal care, school food policies, and marketing regulations [5]. At the cornerstone of this approach is the no harm notion; addressing one form of malnutrition should not be detrimental to subsequently tackling another type of malnutrition at the other end of the spectrum. 

The objectives of this scoping review are two-fold: firstly, to synthesize literature on interventions explicitly tackling DBM and describe the current use of DDAs since the term has been coined, and secondly, to outline epidemiological, clinical and operational research gaps that will need to be addressed to inform future policies.

## 2. Methods 

Distinct to systematic reviews, scoping reviews generally address broader research questions and can include studies of different methodological designs. Furthermore, given the study design of scoping reviews, their results are not amenable to a meta-analysis. The methods were organized according to the six following stages: 

### 2.1. Search Strategy

A broad search strategy using the terms “((dual OR double) burden malnutrition) AND (intervention* OR strateg* OR polic*)” was performed in PUBMED without time restrictions. 

### 2.2. Inclusion Criteria 

Article selection criteria included any intervention with methodologically-sound, well described study designs or policy tools or strategies specifically mentioning and addressing the DBM.

### 2.3. Exclusion Criterion 

Articles were excluded if the interventions, or the evaluations thereof, were not designed or did not explicitly mention DBM. 

### 2.4. Identification of The Research Question.

The general research question this scoping review aims to answer is: What types of interventions or evaluations have been carried out to explicitly tackle the DBM? 

### 2.5. Study Selection 

We considered the following four components (Population, Intervention, Comparison, Outcome (PICO)) to assess and categorize studies to be included in this review. 

#### 2.5.1. Population 

The population of any age (children and adults) and geographical origin presenting at least two forms of malnutrition.

#### 2.5.2. Intervention 

The interventions and evaluations examined in this study are those aiming to tackle DBM, as well as both field and theoretical (modelling) studies with the same aim, in a population including both children and/or adults. 

#### 2.5.3. Comparison 

Pre and post interventions (with and without a control group), comparisons, or post intervention cross sectional studies were included, along with modelling studies.

#### 2.5.4. Outcome(s) 

Change in nutritional status measured by anthropometry (e.g., height-for-age z scores, weight-for-age z scores, weight-for-height z scores, stunting, underweight, wasting, body mass index, overweight, obesity, waist-to-height ratio, and central obesity) and/or clinical parameters (e.g., blood pressure, plasma lipids, plasma glucose and insulin, and biomarkers of micronutrient deficiency).

### 2.6. Extraction of Data

To extract data from the selected articles, Microsoft Excel 2010 as used to gather information on author(s), year of publication, study location, study population, aim of the study, study design and method, description of strategy, key findings, and conclusion. Two authors independently completed the data extraction process during review of all selected articles and consensus reached for all eligible studies. All reference lists of retrieved articles were hand-searched for identification of other potential articles. Articles were title-screened and then abstract-screened. Articles that appeared as relevant from the first screening were read in full.

### 2.7. Synthesis of Findings

The interventions were identified by an iterative process of data collating and key findings were broken down into specific categories, derived from the articles rather than a predefined framework. We followed the Preferred Reporting Items for Systematic Reviews and MetaAnalyses—Extension for Scoping Reviews (PRISMA-ScR) checklist and guidelines to ensure a robust and replicable process (Table A1) [6]. 

## 3. Results

**Figure 1:**
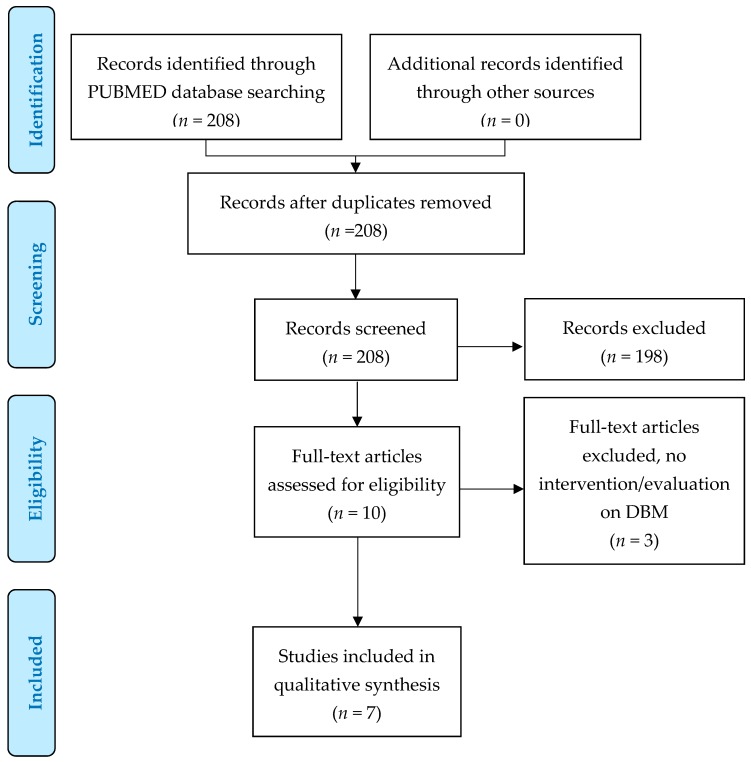
Preferred Reporting Items for Systematic Reviews and MetaAnalyses (PRISMA) flow diagram.

Up until December 2019, we retrieved 208 articles. After title/abstract screening, 11 (5.2%) were eligible for full text screening. Of these, seven studies were eligible for this scoping review (Figure 1). Articles were published between 2015 and 2019. The main characteristics of the interventions selected for review are summarized in Table 2 and described below.

### 3.1. Study Population

Studies concerned three interventions involving school children (range 8 to 14 years), three on women (two on mothers, one on non-pregnant women), and one family intervention. The sample size ranged from 68 to 1350. 

### 3.2. Study Design

Evaluations were of heterogeneous design. Only one study was a randomized control trial, two were pre-post studies, two were post-intervention studies, and finally two were evaluations based on modelling.

### 3.3. Geographical Location

Three studies took place in sub Saharan Africa (Burkina Faso, Ghana, and South Africa), one in Central America (Guatemala), two in Southeast Asia (Indonesia), and one modelling study included 20 low-income countries.

### 3.4. Key Findings

Double-duty actions include interventions, programs, and policies that have been proposed as effective strategies to simultaneously reduce the risk or burden of both undernutrition and overweight, obesity, or diet-related NCDs. Our scoping review identifying and characterizing interventions based on this concept revealed only a small and heterogeneous group of interventions and evaluations that can be divided according to their scope. There were two studies which explored the cost-effectiveness of a policy strategy through modelling, two studies on the impact of national food fortification programs, two studies on the impact of school-based programs, and one on the impact of a behavioral intervention on mothers.

#### 3.4.1. Local, Regional, and National Policies 

Based on linear programming, which is considered to be a useful tool in developing countries for constructing nutritious and health-promoting diets optimized for cost-efficiency [7,8,9], Nykanen and coworkers [10] created a culturally acceptable food basket tailored to both urban and rural settings, which were also optimized in terms of costs and nutrient recommendations. In addition, these food baskets could potentially reduce the prevalence of micronutrient deficiencies by including more nutrient-dense foods that are rich in iron, folic acid, vitamin A, calcium, riboflavin, niacin, and vitamin B12, and foods rich in polyunsaturated fatty acids.

The optimal food basket containing 13 items, satisfied some of the recommended daily nutritional requirements to redress the low uptake of iron, iodine, vitamin A and folic acid encountered in Ghana and at the same time help prevent obesity and diet-related NCDs [10].

#### 3.4.2. Regional Changes in Minimum Wage

Conklin and coworkers [11] developed separate multilevel models to estimate the impact of a US $10 increase in monthly minimum wage in 24 low-income countries and reported a significant association with an accelerated decline in women’s underweight prevalence but observed no impact on the pace of growth in obesity prevalence [11]. 

#### 3.4.3. National Food Fortification Programs 

Two post-intervention studies of national food fortification programs have been identified to specifically target DBM. Faber and coworkers [12] conducted a post-intervention study involving two rural areas (*n* = 346) and two urban areas (*n* = 401) in South Africa. As rural children were found to benefit more than urban children from the three national vitamin A supplementation campaigns held in 2008, 2009, and 2010, the authors recommended that provincial differences in prevalence of vitamin A deficiency should be considered to prevent excessive vitamin A intakes. In this line, Bielderman and coworkers [13] found similar results in a post-intervention study using a convenience sample of pregnant and lactating women (*n* = 234) in the Western Highlands of Guatemala where the national food fortification programme (vitamin A) was maintained for over two decades. The programme had not benefitted a proportion of pregnant and lactating women in rural settings. Whilst rural women not meeting their status-specific vitamin A were found to be 3.5 times more prevalent than urban women, in the urban area, 26 women (21%) had preformed vitamin A intakes above 1500 μg on the day of data collection [13].

#### 3.4.4. School-Based Interventions

School-based nutrition programs, consisting of multi-component interventions including promoting healthy eating, less sedentary behaviour, and more physical activity have been increasingly implemented worldwide since WHO launched the Nutrition-Friendly School Initiative (NFSI) in 2006 to address the double burden of nutrition-related ill health among school-aged children [14]. Edde and coworkers [15] undertook a baseline study to explore the impact of a NFSI intervention in Burkina Faso comparing six intervened schools and six control schools matched at baseline, with a total sample of 699 and 651 pupils in 2009 and 2014, respectively. A modest role for the NFSI in reducing thinness was observed, and although there was no impact on overweight, the authors suggested that further long-term assessment will be needed to observe the impact of the improved nutrition-related practices among children [15]. In a sample of 68 elementary schoolchildren in Rural West Java, Indonesia, Sekiyama and coworkers [16] evaluated a locally sustainable school lunch intervention addressing DBM, and specifically to improve hemoglobin and hematocrit levels and BMI. After the intervention, both hemoglobin and hematocrit levels were significantly improved, thereby almost halving the rate of anemia. Whereas body mass index (BMI) significantly increased in the baseline underweight/normal group, no impact on reducing BMI in the overweight/obese group was observed three months later [16].

#### 3.4.5. Behavioural Interventions Targeting Mothers

Behavioral interventions are often developed to improve modifiable behaviors contributing to malnutrition and to equip mothers with the skills necessary to overcome these problems. Mahmudiono and coworkers [17] carried out a randomized control trial on households in urban Indonesia experiencing DBM in the form of the coexistence of stunted children and overweight/obese mothers. A total of 71 mother–child pairs were randomly assigned to receive either a 12-week nutrition education and home visits or printed educational materials, in addition to a government-funded food supplementation program. Although there was a positive effect of time in children’s weight and height in both groups, no difference was observed between intervention groups. A significantly stronger improvement in maternal self-efficacy to engage in physical activity, consume of fruits and vegetables, and provide children with growth-promoting animal protein was observed among the 12-week education group. No impact was detected on maternal primary outcome measures such as weight, waist circumference, and BMI [17].

## 4. Discussion

### 4.1. Synthesis of Findings

Our scoping review reveals a scarcity of studies reporting interventions simultaneously tackling undernutrition and overweight and obesity (DBM); surprisingly, no study explicitly mentioned DDAs. Although much guidance has been proposed lately, for example in WHO policy briefs [3], the latest Global Nutrition Report (2018) [18], and recently in UNICEF’s report on the State of the World [19], our review yielded very few studies or interventions explicitly targeting DBM through the five established WHO double-duty actions. 

From the set of WHO’s recommended DDAs, (Table 1) no intervention on regulations on marketing was identified. This was despite their potential to impact DBM through their high percentage of environmental sustainability-shared drivers (62.5%) [20]. School-based food programs, which the DDA identified to yield the second largest impact (45.8%) [20], were the focus of only two studies [15,16]. These interventions found differential outcomes and a significant impact on reducing thinness, with nevertheless no impact on overweight/obesity of school-aged children in Burkina Faso and Indonesia. However, it is noteworthy that the sample size and follow up time may not have been optimal to detect significant differences in anthropometric measures [16]. Whilst no intervention on maternal nutrition and antenatal care program (a DDA proposed by WHO) has been identified, a behavioral intervention study targeting mothers [17] was retrieved. Surprisingly, no studies on DDAs protection and promotion of exclusive breastfeeding nor actions to optimize early malnutrition were identified. 

Akin to the two school interventions, this study [17] underscores how interventions targeting simultaneous forms of malnutrition have been less successful at tackling overweight/obesity. While a borderline significant weight increase was observed among underweight children, the intervention failed to have an impact on reducing waist circumference and BMI in overweight or obese mothers. However, the time period to assess the effectiveness of these behavioural interventions may not have been of sufficient duration to detect any significant effects on maternal anthropometric measures.

Straying from the established DDA framework, are four studies exploring national food fortifications programs and policy modelling. Two studies in Guatemala and South Africa, countries with high cultural and sociodemographic diversity, evaluated the impact of national food fortification programmes on the DBM. These two studies highlighted the differential impact of national programmes and underscored the need for context-specific, tailored interventions to eradicate vitamin A deficiencies to prevent underconsumption of the vitamin as well as its excessive intake, which can increase the risk of bone demineralisation and fractures [21,22]. Two distinct modelling studies have shown the potential utility two tools—the culturally adapted linear programming tool and the US$10 minimum wage increase—to advise national programs [10,11]. Nevertheless, it is noteworthy that whilst the culturally adapted linear programming tool to design diets at minimum cost for low-income Ghanaian families is expected to address a low intake of micronutrients (iron, iodine, vitamin A, and folic acid) as well as obesity and diet-related NCDs, a poverty-reduction approach consisting of a minimum wage increase was shown to be insufficient to tackle obesity in this modelling study. 

## 4.2. Strengths and Limitations

Scoping reviews are considered when mapping broad topics, especially where an area is complex or has not been reviewed comprehensively before. The strengths of this review include the unravelling of emerging approaches to deal with the increasing DBM in developing countries. Due to the increasing concern of multiple burdens of malnutrition and the existence of guidance from the WHO we seek to understand how the proposed framework of DDAs is currently addressed. The use of the PRISMA-ScR guidelines ensures the robust and replicable process and the use of the conceptual framework to guide reporting and analysis. However, we acknowledge a number of limitations. First, we may have failed to capture potential relevant multidisciplinary interventions tackling the DBM, which may not have applied this specific nomenclature, despite the surge of interest within the global nutrition community. Second, as we restricted ourselves to peer reviewed literature published in English indexed in PUBMED, we may have not captured relevant studies published in other languages and research published in government reports Third, in light of the scarcity of evaluated interventions, the heterogeneity between interventions, as well as the wide range of study designs included in the scoping review, the direct extrapolation to other socio-economic settings may be precluded.

## 4.3. Public Health Impact of Findings 

The findings of our review noting how a poverty-reduction approach may tackle underweight and low intake of micronutrients is in line with observations of a recent review where family income was positively associated with healthy food consumption in preschool-aged children [23]. Although policy addressing DBM entails numerous complexities, our review on the currently available literature underscores that national policies in culturally diverse countries need to appreciate regional differences to prevent excessive vitamin intakes among certain populations while simultaneously reaching the nutritionally vulnerable. Also, it is of paramount importance that the impact of addressing one form of malnutrition does not exacerbate another type of concomitant or subsequent form of malnutrition. For instance, in Thailand, while a Poverty Alleviation Plan aiming at achieving basic minimum needs was successful in reducing child malnutrition, the subsequent nutrition education interventions targeting the emerging burden of overweight and obesity have not met with the same level of success [24]. Similarly, in industrialized countries, efforts to tackle obesity have been shown to potentially induce emerging nutrition disorders. A recent study in Poland suggested how health promotion activities to control overweight and obesity have inadvertently resulted in an increase of women suffering from nutritional-related disorders, underscoring the need to differentiate health promotion interventions to control overweight and obesity according to gender and age [25]. Furthermore, as a positive association has been suggested between intake of animal flesh food and iron status of adults within developed countries [26], the dietary recommendations to reduce or stop meat intake in developed countries may result in a higher prevalence of anemia among women of reproductive age [27].

In view of the various facets of malnutrition depending on the population, it may therefore be more accurate to speak of multiple burdens of malnutrition. Moreover, it is pivotal to fully discern the impact that globalization has had on rapid urbanization and its attendant shift from a traditional diet to a Western diet, along with an appreciation on how these changes may have conspired in giving rise to the obesity epidemic. Whilst literature suggests that in low- and middle- income countries nutrient deficiencies can be improved through the implementation of poverty reduction measures, a three-pronged approach may be needed to curtail obesity, including nutrition education, promotion of physical activity, and the encouragement of traditional dietary habits. One example of how the use of local food policies defined by civil society and government can be availed of to promote traditional diets with a view to combatting the DBM is the Go Local awareness campaign in Federate States of Micronesia [28].

## 4.4. What is The Way Forward? 

In light of the limited funding of US $31 millions channeled towards obesity and NCD prevention and control highlighted in the 2018 Global Nutrition Report, interventions must build on current strategies and yet be innovative [18]. As evidence is accumulating that both undernutrition and infection early in life may predispose to overweight later in life (which may set in motion a surge of metabolic diseases susceptible to progression due to subsequent infections), synergies need to be exploited. This may be achieved by integrating interventions, promotion of traditional diets, physical activity, and health literacy within a wider infectious disease and poverty reduction framework. Similarly, the potential role of social programs to serve as an entry point for monitoring lifestyle conditions and enhancing access to primary health care of vulnerable children affected by the DBM, especially in high-income countries, should be explicitly recognized [29]. 

To this end, the advent of global accessibility of social media, in conjunction with the emergence of public–private partnerships, offer a window of opportunities which may be harnessed to reach parents, families, and communities. However, a multisectoral response in its own right may not be a silver bullet as its capacity to tackle the multifaceted nature of DBM will depend on institutional and infrastructure support, adequate intersectoral coordination and political will.

## 4.5. Epidemiological and Operational Research Gaps

### 4.5.1. Epidemiological Research Gaps

(1) There is a need for more disaggregated data on the size of the DBM by urban, peri urban, and rural areas to design tailored interventions. Furthermore, in light of the growing number of migrants and internally displaced persons at a global scale, disaggregated data on the nutritional status of these populations with a view to designing appropriate public health interventions to eliminate inequities are warranted. 

(2) There is a need for pre-post interventions to test the cost effectiveness of culturally sensitive linear programming already developed in some countries with initially promising results and test the transferability of this approach to other socio-economic settings. 

(3) Multisectoral action may work best given the complex nature of prevailing circumstances in urban poor settings, however further research is needed to elucidate the different pathways to the coexistence of overweight/obesity and undernutrition.

### 4.5.2. Operational Research

(1) There is a need for more interventions related to all five WHO proposed DDAs, which would provide a deeper understanding of how interventions can be finetuned for enhanced effectiveness.

(2) Whilst the WHO encourages governments, health jurisdictions, and civil society to engage with the private sector through public–private partnerships to address malnutrition [30], the exact role of the private sector needs to be determined when operating with governments, family, schools, communities, policymakers, Non Governmental Organizations, and the media.

(3) The feasibility and effectiveness of context-specific multisectoral interventions need to be explored according to the type and degree of decentralized structure in place. 

## 5. Conclusions

Despite the growing problem of DBM, there is a dearth of studies examining DDAs as proposed by the WHO. The few studies retrieved in this review illustrate the complexity of the issue at hand and the pivotal importance of interventions appreciating regional differences in dietary habits, which national data often fail to reflect, in order to reach the nutritionally vulnerable and at the same time prevent excessive micronutrient intakes. As a corollary, it can be posited that it would be of importance that the differential impact of globalization on urbanization, migration, and gender be considered and interventions tailored accordingly. While literature suggests that in both low- and middle-income countries as well as high-income countries nutrient deficiencies can be curbed by poverty reduction measures, a three-pronged approach may be required to curtail obesity, one of which would include nutrition education and promotion of physical activity, along with the encouragement of local food production and traditional diets. 

Whilst the DBM constitutes a key challenge to the international community, it also offers a wealth of opportunities for interventions. Seen within its wider context, malnutrition and all its attendant forms lends a critical point for integrated action and a port of entry for coordinated responses to infectious diseases, NCDs, maternal and child illnesses, and diseases associated with ageing. Addressing the DBM should also be seen as a catalyst for mitigating policy challenges beyond health, including economic inequality within populations, poverty, and gender inequity, which should draw upon the combined insights of nutritional, agricultural, clinical, and political sciences.

## Figures and Tables

**Table 1:** The World Health Organization’s recommended double-duty actions to tackle the double burden of malnutrition.

Double-Duty Actions to Tackle the Double Burden of Malnutrition [5]
Promotion and protection of exclusive breastfeeding in the first 6 months Promotion of appropriate early and complementary feeding in infants Maternal nutrition and antenatal care programs Regulations on marketing School food policies and programs

**Table 2:** Summary of the intervention or evaluation studies identified targeting the double burden of malnutrition.

Reference	Year	Country	Study Design	Intervention	Sample Size	Age	Key Findings
Faber et al. [12]	2015	South Africa	Post intervention survey	National vitamin A supplementation program introduced in 2002, targeting children ages 6 to 59 months from low socioeconomic communities	KwaZulu-Natal (*n* = 140) and Limpopo (*n* = 206); an urban area in the Northern Cape (*n* = 194); and an urban metropolitan area in the Western Cape (*n* = 207)	Mean age of the children: 3.7 ± 1.1 years	Large variations in anthropometric status highlight the importance of targeting specific nutrition interventions, taking into account the double burden of overnutrition and undernutrition
Bielderman et al. [13]	2016	Guatemala	Post intervention survey	Population-wide vitamin A fortification of table sugar program spanning two decades	Convenience sample of 234 pregnant and lactating women in the Western Highlands	Mean age of the mothers: 24 ± 6 years	There was a differential impact of the national program; rural women not meeting their status-specific vitamin A were 3.5 times more common than urban women. In the urban area, 26 women (21%) had vitamin A intakes above 1500 μg on the day of data collection
Sekiyama et al. [16]	2017	Indonesia	Pre-post study	One-month school lunch intervention aiming at sustainability and based on children’s nutritional intake, hemoglobin and hematocrit levels, and body mass index (BMI)	68	114.6 months ± 8.8 (fourth grade)	After the intervention, hemoglobin (*p* < 0.05) and hematocrit (*p* < 0.05) levels were significantly improved. BMI significantly increased in the baseline underweight/normal group (*p* < 0.001) but not in the overweight/obese group
Mahmudiono et al. [17]	2018	Indonesia	Randomized controlled trial	Behavioral intervention (including home visits) coupled with a government food supplementation program and educational materials for children	Seventy-one mother–child pairs	39.57 months in intervention group (Neo Mom group) and 40.24 months (control group)	Strong improvement in maternal self-efficacy to engage in physical activity, eat fruits and vegetables, and to provide children with growth-promoting animal protein, but no significant influence on child height gain
Nykänen et al. [10]	2018	Ghana	Modelling study	Calculations included implementing cultural acceptability for families living in extreme and moderate poverty	NA	NA	Using culturally acceptable food baskets (FB) of minimum cost could be developed for urban and rural low-income Ghanaian families for a month when assuming that half of the household income is spent on food. These FBs would fulfil all nutrient recommendations, and at the same time can help to prevent obesity and diet-related non-communicable diseases (NCDs).
Conklin et al. [11]	2018	Twenty-four low-income countries	Modelling study	Examine changes in minimum wage associated with changes in women’s weight status	NA	NA	A US $10 rise in monthly minimum wage significantly accelerated the decline in women’s underweight prevalence, but had no association with the pace of growth in obesity prevalence.
Edde et al. [15]	2019	Burkina Faso	Pre and post interventions with controls	Nutrition-Friendly School Initiative (NFSI)	699 and 651 pupils in 2009 and 2014, respectively	2009: 8 to 11 years (454 or 70 %); 12 to 14 years (195 or 30%)	A modest role for the Nutrition-Friendly School Initiative in reducing thinness, but not overweight

## Appendix A

**Table A1:** PRISMA-ScR checklist [6].

SECTION	ITEM	PRISMA-ScR CHECKLIST ITEM	REPORTED ON PAGE #
TITLE			
Title	1	Identify the report as a scoping review.	1
ABSTRACT			
Structured summary	2	Provide a structured summary that includes (as applicable): background, objectives, eligibility criteria, sources of evidence, charting methods, results, and conclusions that relate to the review questions and objectives.	2
INTRODUCTION			
Rationale	3	Describe the rationale for the review in the context of what is already known. Explain why the review questions/objectives lend themselves to a scoping review approach.	4
Objectives	4	Provide an explicit statement of the questions and objectives being addressed with reference to their key elements (e.g., population or participants, concepts, and context) or other relevant key elements used to conceptualize the review questions and/or objectives.	4
METHODS			
Protocol and registration	5	Indicate whether a review protocol exists; state if and where it can be accessed (e.g., a Web address); and if available, provide registration information, including the registration number.	No protocol exists
Eligibility criteria	6	Specify characteristics of the sources of evidence used as eligibility criteria (e.g., years considered, language, and publication status), and provide a rationale.	4
Information sources	7	Describe all information sources in the search (e.g., databases with dates of coverage and contact with authors to identify additional sources), as well as the date the most recent search was executed.	4
Search	8	Present the full electronic search strategy for at least one database, including any limits used, such that it could be repeated.	4
Selection of sources of evidence	9	State the process for selecting sources of evidence (i.e., screening and eligibility) included in the scoping review.	5, and Figure 1
Data charting process	10	Describe the methods of charting data from the included sources of evidence (e.g., calibrated forms or forms that have been tested by the team before their use, and whether data charting was done independently or in duplicate) and any processes for obtaining and confirming data from investigators.	5
Data items	11	List and define all variables for which data were sought and any assumptions and simplifications made.	5
Critical appraisal of individual sources of evidence	12	If done, provide a rationale for conducting a critical appraisal of included sources of evidence; describe the methods used and how this information was used in any data synthesis (if appropriate).	
Synthesis of results	13	Describe the methods of handling and summarizing the data that were charted.	5
RESULTS			
Selection of sources of evidence	14	Give numbers of sources of evidence screened, assessed for eligibility, and included in the review, with reasons for exclusions at each stage, ideally using a flow diagram.	Figure 1
Characteristics of sources of evidence	15	For each source of evidence, present characteristics for which data were charted and provide the citations.	Table 1
Critical appraisal within sources of evidence	16	If done, present data on critical appraisal of included sources of evidence (see item 12).	
Results of individual sources of evidence	17	For each included source of evidence, present the relevant data that were charted that relate to the review questions and objectives.	5–8, and Table 1
Synthesis of results	18	Summarize and/or present the charting results as they relate to the review questions and objectives.	Table 1
DISCUSSION			
Summary of evidence	19	Summarize the main results (including an overview of concepts, themes, and types of evidence available), link to the review questions and objectives, and consider the relevance to key groups.	8–9
Limitations	20	Discuss the limitations of the scoping review process.	10
Conclusions	21	Provide a general interpretation of the results with respect to the review questions and objectives, as well as potential implications and/or next steps.	10–13
FUNDING			
Funding	22	Describe sources of funding for the included sources of evidence, as well as sources of funding for the scoping review. Describe the role of the funders of the scoping review.	No sources of funding
